# A module-based approach for post-omics, post-GWAS network-based gene classification

**DOI:** 10.1093/bioinformatics/btag571

**Published:** 2026-07-31

**Authors:** Alexander McKim, Christopher A Mancuso, Arjun Krishnan

**Affiliations:** Department of Biomedical Informatics, University of Colorado Anschutz, Aurora, CO 80045, United States; Department of Biostatistics and Informatics, Colorado School of Public Health, University of Colorado Anschutz, Aurora, CO 80045, United States; Department of Biomedical Informatics, University of Colorado Anschutz, Aurora, CO 80045, United States

## Abstract

**Motivation:**

Complex traits and diseases are highly polygenic and understanding the full set of genes involved is a central challenge in biomedicine. However, due to sample size limitations and noise (technical and biological), experimental approaches for disease-gene discovery such as transcriptomics and GWAS result in long, noisy, heterogeneous gene lists, which may be trimmed to a subset of likely relevant genes while leaving several false negatives. Computational gene classification approaches, especially those using genome-scale molecular interaction networks, are promising avenues for complementing such experimental findings by analytically expanding observed gene lists based on the functional relatedness between genes. We previously introduced the network-based gene classification approach, *GenePlexus*, which was rigorously benchmarked to show state-of-the-art performance, especially for predicting novel genes associated with biological processes and fine-grained phenotypes. Network-based gene classification performance,however, declines for diseases, especially when the inputs are omics and GWAS-based long gene lists.

**Results:**

Here, we show that these disease gene lists span multiple biological processes spread across the molecular network, and we propose *ModGenePlexus*, a new network-based gene classification method that takes a two-stage approach. First, clustering and semi-supervised learning decomposes the input gene list into coherent, denoised network gene modules. Then, *ModGenePlexus* trains supervised (*GenePlexus*) classifiers for each module and aggregates predictions to return genome-wide rankings. We benchmarked *ModGenePlexus* across simulated data, transcriptomic signatures, and GWAS datasets (together spanning hundreds of diseases), showing improved recovery of known disease genes compared to *GenePlexus*. Beyond improved classification, the results of enrichment analysis of *ModGenePlexus* outputs are much more interpretable by virtue of revealing nuanced biological processes. Together, these results establish *ModGenePlexus* as a scalable, interpretable tool for gene classification of GWAS- and omics-derived gene lists across diverse biological contexts.

**Availability and implementation:**

*ModGenePlexus* is freely available on GitHub at https://github.com/krishnanlab/ModGenePlexus, and the full source code and results supporting this study are available on Zenodo at https://zenodo.org/records/19857910.

## 1 Introduction

Determining the full complement of genes that play a role in a given cellular pathway, fine-grained (developmental, anatomical, behavioral) phenotype, or complex trait/disease is a grand challenge in the fields of genetics and molecular biology. In the case of pathways and phenotypes, careful experimental discovery is still the gold standard for addressing this challenge, but it is labor intensive and is not scalable across the genome. Therefore, computational gene classification methods have emerged as critical complements that enable researchers to automatically prioritize pathway/phenotype gene annotations (Guan *et al.* 2010, Warde-Farley *et al.* 2010, Lee *et al.* 2011, Wang *et al.* 2011, Leiserson *et al.* 2015, Krishnan *et al.* 2016, Liu *et al.* 2020b, Mancuso *et al.* 2024) for experimental follow-up. These methods aim to expand known gene sets by identifying novel genes likely to share functional roles, helping address the question: *what other genes are involved in my pathway/phenotype of interest?*

Because a gene’s role in a pathway/phenotype emerges from its interaction with other genes, network-based gene classification methods, which leverage the functional relationships (i.e. molecular network connections) among genes to predict novel pathway/phenotype annotations, have proven widely successful for this task (Kim *et al.* 2022, Zitnik *et al.* 2024). Our group previously developed *GenePlexus* (Liu *et al.* 2020b, Mancuso *et al.* 2023), a network-based approach that builds a supervised machine learning (ML) model trained on the network relationships of known genes and uses this model to classify novel genes, with each gene associated with a probability based on the similarity of its global network connectivity to that of the known gene list. Based on extensive benchmarking across diverse gene sets of cellular pathways, diseases, and traits, we showed that this supervised learning approach outperforms the classical label propagation method that spreads information from known genes along the network edges to infer novel genes (Šubelj 2019). *GenePlexus* performs well when applied to smaller, coherent (highly inter-connected) gene sets such as biological processes and fine-grained phenotypes that are highly localized within a single neighborhood in the genome-scale network (Luo *et al.* 2025, Yuan *et al.* 2025).

**Figure 1:**
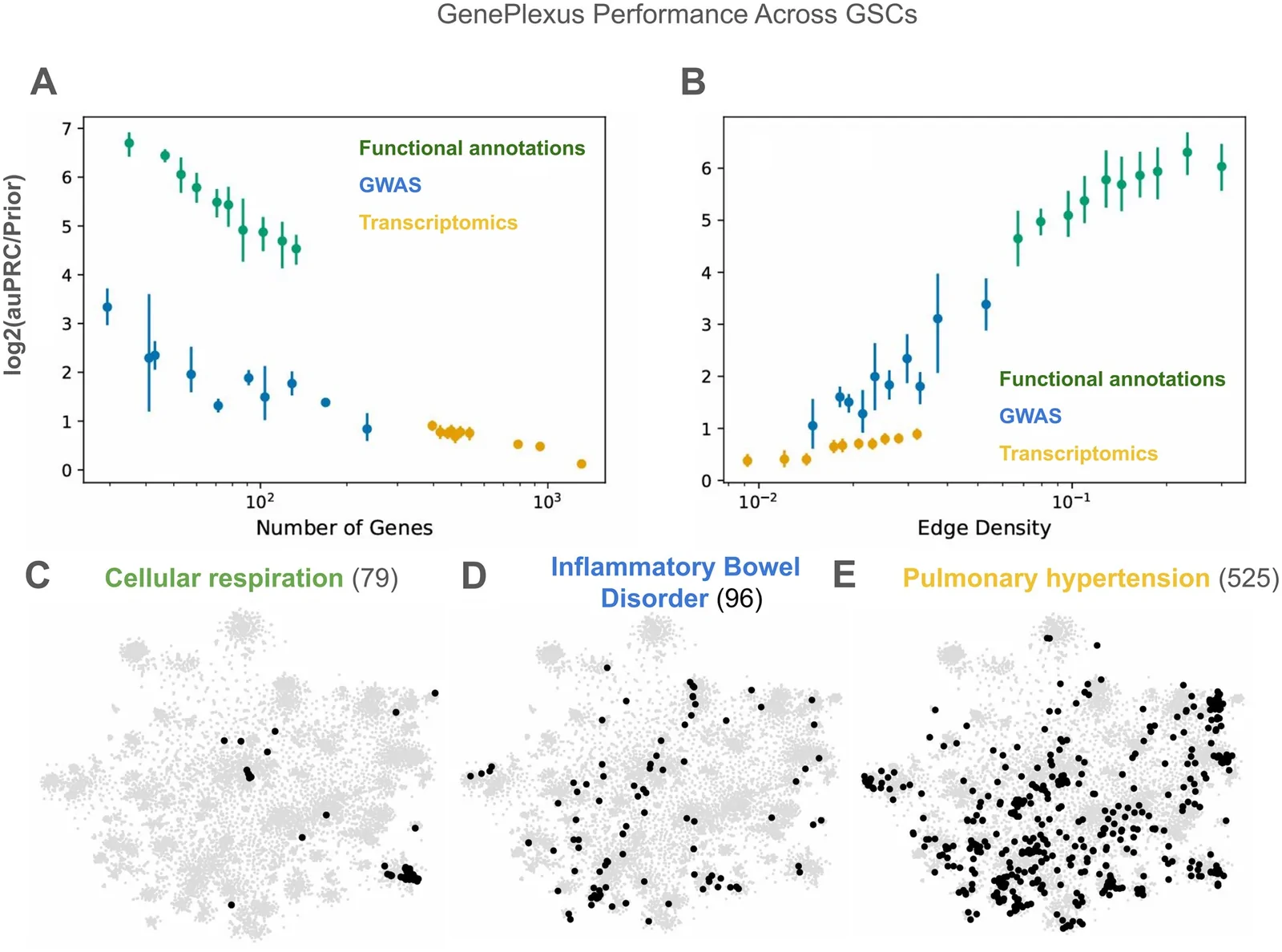
GenePlexus performs poorly on large, dispersed gene sets. (A) GenePlexus performance as a function of gene set size across different data modalities. Larger gene sets, particularly from transcriptomic datasets, exhibit consistently poor performance. (B) GenePlexus performance as a function of network edge density across data modalities. Despite increased edge density in transcriptomic-derived gene lists, performance does not improve relative to other modalities, highlighting its limitations in large, complex gene sets. (C) The localization of genes of an average size biological process. (D) The genes of an average sized GWAS set. The genes are not as localized within the network. (E) The genes of an average sized, manually curated gene list in the CREEDS disease transcriptomics data.

However, the performance of *GenePlexus* degrades for gene sets that contain large numbers of genes, which are precisely the kinds of gene sets that correspond to complex diseases, typically recovered from the two most widely-used empirical disease-gene finding approaches: transcriptomic profiling (omics) and GWAS. While powerful, these two approaches can generate *large* (100s to 1000s of genes/SNPs), *noisy* (false positives/negatives) and *heterogeneous* (genes contributing to many diverse pathways/processes) gene sets (Kaler and Purcell 2019, Buzdugan *et al.* 2016). The noise in omics and GWAS studies can stem from many different sources such as low sample size, small effect size (with small sample and effect size leading to low power), technical noise, and biological heterogeneity of the samples (Schurch *et al.* 2016, Degen and Medo 2025). Typical network-based gene classification methods, like *GenePlexus*, are powerful techniques to overcome the noise stemming from false negatives, but performance suffers when false positives are present (Fig. 1). An additional characteristic of omic and GWAS gene sets of complex traits and diseases is that the underlying biology is often multi-factorial, resulting from perturbations of a diverse set of processes and pathways leading to functionally heterogeneous gene sets. In the context of a genome-wide molecular network, these processes/pathways form tightly clustered modules that localize in different neighborhoods throughout the network, limiting the effectiveness of methods like *GenePlexus* that look for a highly localized network pattern corresponding to a single neighborhood.

To address these challenges, we propose *ModGenePlexus*, a novel network-ML approach that: (i) takes a divide-and-conquer approach, first by breaking a large input list into smaller, highly connected “modules” and then (ii) utilizing the powerful *GenePlexus* methodology to make module-specific predictions, which are later reconciled into a master predicted gene list. This first step helps account for the multi-factorial nature (Barabási *et al.* 2011) of complex disease gene sets as well as tackles the problem of false positives, which are removed by virtue of these genes being more loosely connected in the network to the rest of the disease genes. The second step can remove false negatives by predicting biologically relevant genes that were missed in the omics or GWAS gene set. Ultimately *ModGenePlexus* takes advantage of gene networks, capturing biological processes and pathways as local network neighborhoods (Gustafsson *et al.* 2014), while refining the initial, large input gene lists through clustering into coherent modules.

We validated *ModGenePlexus* through a series of systematic evaluations using a study-bias holdout validation scheme (*see Materials and methods*) on large, simulated and real-world datasets to demonstrate its robustness in diverse gene classification scenarios. First, we conducted simulations using “traits” constructed by combining multiple biologically meaningful gene sets to assess performance for varying gene set sizes and noise levels. These simulations confirm that *ModGenePlexus* outperforms *GenePlexus*, particularly in settings with heterogeneity or false positive genes. We then applied *ModGenePlexus* to differentially-expressed genes from hundreds of public transcriptomic datasets (Wang *et al.* 2016) and demonstrated substantial performance improvements over *GenePlexus* across a wide range of diseases, drug treatments, and gene perturbations. Next, we evaluated *ModGenePlexus* on GWAS data, demonstrating that it accurately predicts significantly associated genes, indicating its ability to navigate the inherent noise and redundancy in GWAS-derived gene lists. Finally, to illustrate the interpretability and added value of *ModGenePlexus*, we present a case study on type 2 diabetes, which demonstrates how its inherent modular approach provides insight into distinct gene modules, uncovering processes missed by non-modular (single-model) classification applied to traits with highly complex genetic architecture. Together these results highlight the advantages of a module-based approach for gene classification with large, noisy, heterogeneous gene lists from high-throughput experiments and emphasize the broader implications of network-based modularity for interpreting and generating novel hypotheses from complex biological datasets generated by large-scale -omics studies.

## 2 Materials and methods

### 2.1 Data processing

#### 2.1.1 Network processing

To cluster and to generate network-based features for the supervised learning model we leveraged the widely-utilized STRING (Szklarczyk *et al.* 2015) functional network throughout this work. We downloaded v10 of the *Homo sapiens* network (*9606.protein.links.detailed.v10.0.txt*) directly from the STRING website and retained only edges with an edge weight >0.7. The Ensembl protein IDs were mapped to Entrez IDs using mygene.info (Wu *et al.* 2013), and in the case where one Ensembl protein ID mapped to more than one Entrez ID we added an edge for each Entrez ID. This resulted in a network with 16 624 nodes and 400 729 edges. The original *GenePlexus* paper (Liu *et al.* 2020b) has systematically demonstrated that the full combined STRING network outperforms the version with experimentally validated edges alone, which suggests that integrating evidence types collectively captures functional relationships better than other network configurations. These evidence types include physical interactions, coexpression, gene annotation databases, etc.

#### 2.1.2 Processing gene set collections

We evaluated *ModGenePlexus* using a diverse set of gene sets, incorporating Gene Ontology Biological Processes (GOBP) in our simulations and gene sets from CRowd Extracted Expression of Differential Signatures (Wang *et al.* 2016) (CREEDS) and GWAS Atlas (Tian *et al.* 2020) for real-world validation. Gene set size thresholds were chosen to reflect the intended role of each collection. For GOBP, used in simulations, we retained smaller, specific terms (10–100 genes) to allow controlled combinations of distinct biological processes. For CREEDS and GWAS Atlas, which we use for real-world validation of *ModGenePlexus*, we required larger sets (at least 100 genes) to represent sufficient multi-factorial complexity of actual traits and diseases. A common processing step for all gene set collections involves removing genes not found in the processed STRING network. More in-depth information on these gene set collections is provided below and in File 1, available as supplementary data at *Bioinformatics* online.

#### 2.1.3 Gene ontology biological processes

The Gene Ontology Biological Process (Ashburner *et al.* 2000, The Gene Ontology Consortium *et al.* 2023) (GOBP) gene set collection was compiled using gene annotations from mygene.info (Wu *et al.* 2013, Xin *et al.* 2016, Lelong *et al.* 2022). These direct annotations were then propagated using the hierarchical structure of GO and filtered to only retain genes in STRING. Then, the full collection of gene sets was reduced to a broadly non-redundant set of GOBP terms (based on gene set overlap, see File 1, available as supplementary data at *Bioinformatics* online) and filtered down to those that contain at least 10 genes and at most 100 genes. To simulate complex traits composed of multiple distinct biological processes, we randomly combined 2, 5, and 10 GO term gene annotations to create simulated traits. False positive rates of 0.0, 0.25, 0.50, 0.75, and 1.0 were applied, where the rate represents the percentage of the size of the simulated trait. Genes that were not annotated to any GO term in the simulated trait were added as false positives to evaluate *ModGenePlexus* under varying noise levels.

#### 2.1.4 CREEDS database

We compiled gene sets from the CRowd Extracted Expression of Differential Signatures (CREEDS) database (v1.0), which includes manually curated genes from GEO (Barrett *et al.* 2013). We included only manually curated signatures and retained gene sets with at least 100 genes, ensuring sets are sufficiently complex. We used three distinct data modalities: single gene perturbations (*n *= 972), single drug perturbations (*n *= 234), and diseases (*n *= 311).

#### 2.1.5 GWAS Atlas database

We compiled gene sets from the GWAS Atlas (v20191115) utilizing *MAGMA* (Leeuw *et al.* 2015) gene prioritization scores for three different thresholds: $P<1\times 10^{-2}$ (*n *= 670), $P<1\times 10^{-5}$ (*n *= 32), and $P<1\times 10^{-8}$ (*n *= 15), only retaining sets with at least 100 genes. We chose to create gene set collections for multiple *P*-value thresholds from *MAGMA* because, while it is common to use very strict thresholds for GWAS data due to loss of significance from multiple hypothesis correction and the assumption that these top genes capture all the underlying biology of a trait, it has been shown that even genes determined to be less significant can have biological meaning, especially when accounting for their cumulative effects (Chen *et al.* 2021).

### 2.2 Inner-working of *ModGenePlexus*

#### 2.2.1 Gene set clustering

We used *DOMINO* (Levi *et al.* 2021) to generate the modules used by *ModGenePlexus* because it is one of the best methods for finding “active modules” (Lazareva *et al.* 2021), i.e. subnetworks within a large genome-scale network that capture active molecular processes specific to a condition of interest, probed by the analyzed transcriptomics or GWAS study (Mitra *et al.* 2013). *DOMINO* uses Louvain clustering (Blondel *et al.* 2008) and an application of Prize Collecting Steiner Trees (Johnson *et al.* 2000, Tuncbag *et al.* 2013) to partition the genome-wide network by optimizing modularity. *DOMINO* then filters the initial partitions based on a list of genes and applies label propagation to refine the modules, adding interconnected genes from the network and making them more topologically coherent. This process removes some genes from the initial set through filtering while incorporating additional genes via label propagation.

#### 2.2.2 Validation scheme

To evaluate model performance in a way that reflects the real-world challenge of novel gene discovery, we use a study bias holdout scheme. This evaluation is intentionally stringent, as it tests the ability to predict understudied genes, which tend to have fewer or weaker network connections compared to well-characterized genes (Hoelzemann *et al.* 2024, Saidi *et al.* 2025). For all gene sets, the initial positive genes are those that are originally annotated in the gene set. The selection process then differs between methods:

- *GenePlexus*: All originally annotated genes are positive labels.
- *ModGenePlexus*: Genes contained in a given module after running *DOMINO*.

For each input gene set (trait/disease), we assess gene study bias by counting the number of PubMed abstracts (Brown *et al.* 2015) each gene appears in. Genes in the top two-thirds of PubMed mention frequency were designated well-studied and assigned to the positive training set. The bottom one-third, representing understudied genes, are held out as the positive test set. All models use this same positive training/test split to ensure consistency across evaluations. Negative genes were identified using the approach implemented in *PyGenePlexus* (Mancuso *et al.* 2023). This method defines negative examples as genes not annotated to any gene set in a reference collection that significantly overlaps ($P<5\times 10^{-2}$, one-sided Fisher’s exact test) with the input gene set. This process also identifies a set of neutral genes that are not used in training or evaluation. Negative genes were then randomly divided into training and test sets, maintaining the same training-to-test ratio used for positive genes (see File 1, available as supplementary data at *Bioinformatics* online). Importantly, the negative test set consists only of genes that are consistently identified as negative across all models, including both *GenePlexus* and *ModGenePlexus*, ensuring that no negative test gene is used during training in either *GenePlexus* or *ModGenePlexus*.

*GenePlexus* uses the original training positives directly as input to the supervised classifier, with no intermediate filtering or propagation. *ModGenePlexus* applies a three-stage procedure to the same set of training positives: (i) *DOMINO* partitions the input gene list into topologically coherent modules within the STRING network via Louvain community detection, using only training positives as active genes; (ii) *DOMINO* performs label propagation across the network, which can incorporate additional network genes into each module beyond the original active genes; these added genes are *propagated genes*, and the original training positives that remain assigned to a module after propagation are *DOMINO-retained positives* from the initial gene list. Propagated genes may include negatives, neutrals, or held-out test positives that *DOMINO* independently rediscovers; and (iii) each module is passed to *GenePlexus* as the input gene list for an independent module-specific classifier, with all module members (*DOMINO*-retained positives + propagated genes) serving as supervised positive labels.

#### 2.2.3 Running the supervised learning model

*GenePlexus* and *ModGenePlexus* were run using a modified version of *PyGenePlexus* (v1.0.1) (Mancuso *et al.* 2023) Python package. All models were trained using the STRING network with adjacency matrix-based feature representations. *PyGenePlexus* uses a logistic regression with L2 regularization implemented in scikit-learn for classification.

#### 2.2.4 Aggregating module gene predictions

For *ModGenePlexus*, gene scores across modules were aggregated across all identified modules using the tau score (Lüleci and Yılmaz 2022), defined by the formula:


(1) $$\tau =\frac{\sum_{i=1}^{n}\left(1-{\hat{x}}_{i}\right)}{n-1}\;;\;{\hat{x}}_{i}=\frac{x_{i}}{{\text{max}}_{1\leq i\leq n}(x_{i})},$$

where τ is the tau score, $x_{i}$ is a gene’s rank for module *i*, and *n* is the total number of genes for which *ModGenePlexus* returns predictions.

In *ModGenePlexus*, each module has a specific *GenePlexus* model trained for it independently of all other models. The raw probability scores produced by different models are not calibrated (Wallace and Dahabreh 2012) on a common scale and cannot be directly compared or naively averaged across modules. The tau score addresses this issue by quantifying module specificity of a gene based on its rank across model predictions, rather than relying on raw probabilities. Each gene’s final score was computed by aggregating its predicted rank across all identified modules into the tau score. Genes were then ranked based on these scores to produce the final, unified ranking, and the performance of test genes in this ranked list was compared to the baseline performance of *GenePlexus*.

#### 2.2.5 Evaluation metric

Evaluations were conducted using log2(auPRC/prior), which is the area under the Precision-Recall Curve (auPRC) normalized by the prior probability of a gene having a positive label. auPRC and prior are defined as follows:


(2) $$\text{auPRC}=\sum_{n}\left({\text{Recall}}_{n}-{\text{Recall}}_{n-1}\right){\text{Precision}}_{n},$$

(3) $$\text{Precision}=\frac{\text{TP}}{\text{TP}+\text{FP}},\;\text{Recall}=\frac{\text{TP}}{\text{TP}+\text{FN}},$$

(4) $$\text{prior}=\frac{P}{P+N}=\frac{\text{TP}+\text{FN}}{\left(\text{TP}+\text{FN})+(\text{FP}+\text{TN}\right)},$$

where TP is true positives, FN is false negatives, FP is false positives, and TN is true negatives. P and N are the ground truth labels. This approach accounts for class imbalance, ensuring model comparison remains robust despite differences in positive and negative label distributions (Saito and Rehmsmeier 2015).

#### 2.2.6 GOBP enrichment of gene sets

GOBP enrichment was performed using the *clusterProfiler* (Yu *et al.* 2012) R package. Enrichment was conducted separately for *GenePlexus* and for each individual module model prediction in *ModGenePlexus*. For module-based predictions, genes with a probability of ≥0.80 were considered predicted positives. For *GenePlexus*, due to the larger input sizes, enrichment was conducted using either a .80 probability threshold or a threshold that limited the number of predicted genes to at most twice the original input size, whichever resulted in fewer genes. All enrichment analyses were conducted using the *org*. *Hs.eg.db* (3.20.0) annotation package, with default parameters. Significance was determined using a q-value threshold of 0.05.

#### 2.2.7 Normalized beta coefficients of *(mod)GenePlexus* models

To compare the contribution of the most important features across models, we extracted the raw logistic regression coefficients from the trained *GenePlexus* and *ModGenePlexus* classifiers and created heatmaps using the ComplexHeatmap (Gu *et al.* 2016) and circlize (Gu *et al.* 2014) R packages. To enable direct comparison across models and modules, we applied mean-centered scaling followed by max-absolute-value scaling, normalizing the coefficients within each model to a common range of [−1,1]. Specifically, for each model, each coefficient was centered by subtracting the mean across all coefficients, and then divided by the maximum absolute centered value to facilitate comparison across modules:


(5) $${\text{scaled}}_{i}=\frac{\beta_{i}-\bar{\beta }}{\text{max}(|\beta_{i}-\bar{\beta }|)},$$

where $\beta_{i}$ is the $i^{th}$ beta coefficient in the list of model coefficients, and $\bar{\beta }$ is the mean beta coefficient value. We visualize the top 20 scaled model coefficients across each module and *GenePlexus*, where each coefficient in this case applies to the top 20 gene predictions for each model.

#### 2.2.8 Calculating information content of GOBPs

To assess the specificity of enriched GOBPs, we calculated the information content (IC) (Mazandu and Mulder 2013) for all enriched terms:


(6) $$IC=-{\;\text{log}\;}_{2}\left(\frac{\#\;\text{genes}\;\text{in}\;\text{GOBP}}{\#\;\text{genes}\;\text{in}\;\text{any}\;\text{GOBP}}\right).$$

## 3 Results

### 3.1 Single-model gene classification leads to poor performance for heterogeneous input gene lists

Following our previous work, we reevaluated *GenePlexus* using diverse data sources, including biological process-gene, disease-gene, and trait-gene associations. While *GenePlexus* performs well on curated datasets, its performance significantly declines when applied to experimental datasets such as transcriptomics-derived gene lists (Fig. 1A and B). These gene lists present unique challenges due to noisy signals, where some relevant genes may not exhibit differential expression (false negatives), while some observed changes may not reflect causal or functionally relevant biology (false positives), making robust predictions more difficult. The correlation between edge density and performance reflects this issue (Fig. 1B): gene sets derived from biological processes tend to have high edge densities and are often localized within a local network neighborhood, while gene sets from GWAS (The DREAM Module Identification Challenge Consortium *et al.* 2019) exhibit lower edge densities and are more scattered in the network. Further, despite increased edge density in transcriptomic-derived datasets, performance does not improve relative to other modalities, highlighting the limitations of *GenePlexus’*s single-model approach for large and highly complex gene sets. For network-based methods, the localization of genes within the network is a key predictor of performance. In STRING, biological processes (Fig. 1C) tend to be more localized than disease- or GWAS/transcriptomics-derived datasets (Fig. 1D and E). These limitations and performance drops are not unique to *GenePlexus*; similar patterns of performance decline are observed in other network-based gene classification methods, including the classical label propagation approaches (Liu *et al.* 2020b).

### 3.2 *ModGenePlexus*: a network-based divide-and-conquer ML approach to tackle long, noisy, heterogeneous gene lists

**Figure 2:**
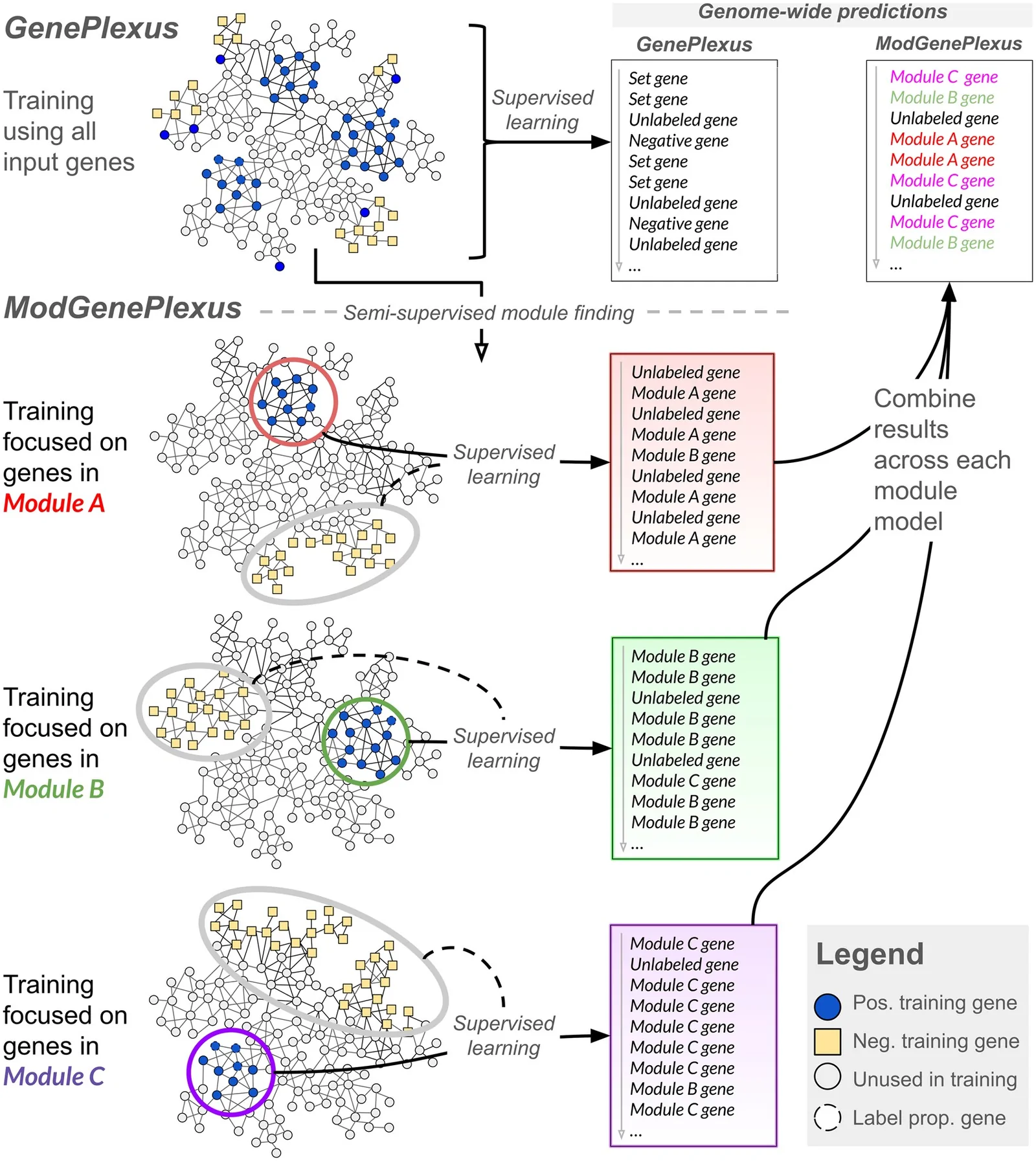
The ModGenePlexus method. Genes are clustered to discover modules, where semi-supervised learning filters out genes that do not belong to sufficiently large modules. Genes outside the original input set but located in disease-enriched modules are incorporated as additional inputs. These refined module gene sets are then processed by GenePlexus, which builds a logistic regression model for each module to classify genes at the module level. The individual module predictions are subsequently aggregated to generate a final gene prediction score.

To address the limitations of single-model gene classification, we developed *ModGenePlexus*, a modular extension of *GenePlexus* designed to improve performance for large, noisy, and heterogeneous gene lists derived from omics experiments (Fig. 2).

*ModGenePlexus* works by first clustering the long list of trait/disease-associated genes into smaller, denoised, topological and biologically coherent subsets that we refer to henceforth as “modules.” *DOMINO*, which has been benchmarked favorably against many competing module discovery methods (Levi *et al.* 2021), performs well at this decomposition task while denoising the input by dropping weakly connected genes and adding new, originally-missed genes via localized label propagation (semi-supervised learning). Finally, network-based gene classification, *i.e. GenePlexus*, is applied independently to each expanded module, finally presenting the top genes for each module and an aggregated top gene prediction across modules. Importantly, the *GenePlexus* scoring step provides an additional layer of prioritization within each module in a genome-wide scoring strategy (Figs 4 and 5, available as supplementary data at *Bioinformatics* online). *ModGenePlexus* improves gene classification accuracy by refining the input gene list by removing weakly connected genes and expanding it to include new candidates with strong module-specific connectivity. This dual refinement is particularly useful for gene lists derived from low-powered and/or minimally preprocessed datasets where false positives and false negatives may be prevalent. We systematically validated *ModGenePlexus* across simulated, transcriptomics-derived, and GWAS-derived gene lists, demonstrating substantial improvements in gene classification performance compared to *GenePlexus*. Additionally, by identifying distinct gene modules, *ModGenePlexus* enhances biological interpretation and reveals context-specific pathways underlying complex traits and diseases.

### 3.3 *ModGenePlexus* accurately recovers genes associated with simulated heterogeneous traits

**Figure 3:**
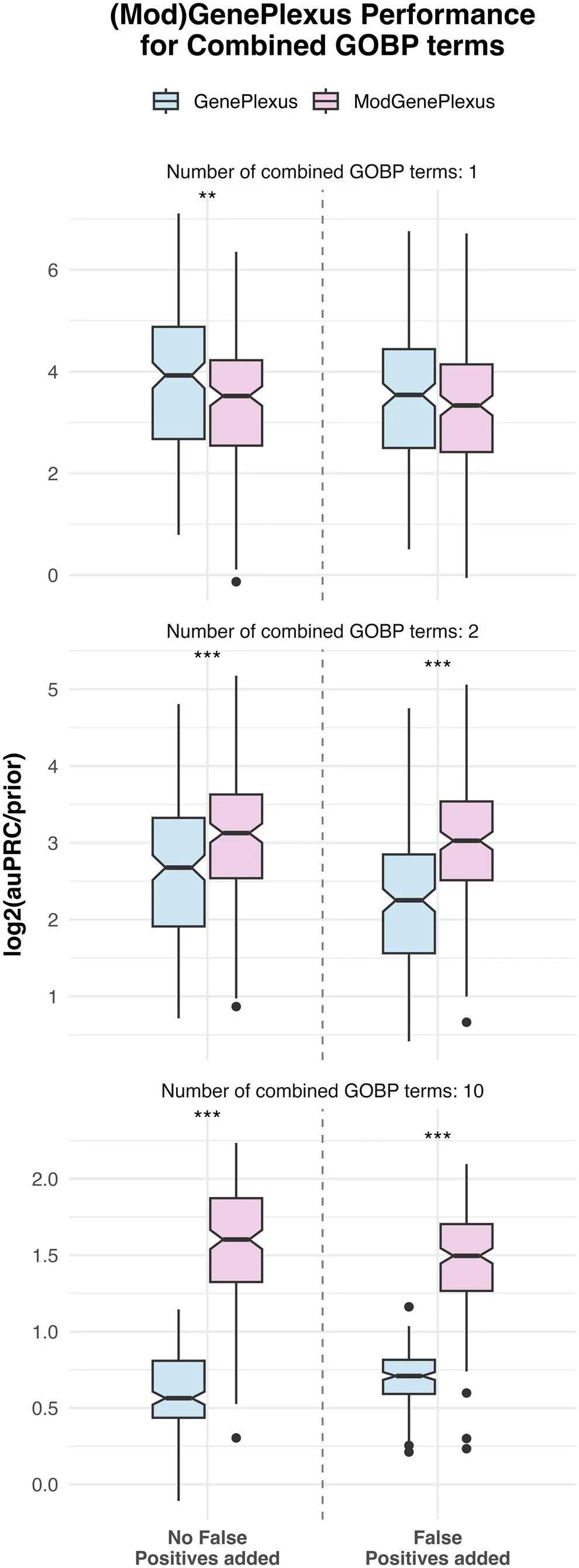
ModGenePlexus outperforms GenePlexus on simulated traits generated by combining multiple GOBP terms. Models were tested on individual GOBP terms and simulated traits composed of 2 or 10 combined terms. False positives were introduced where the number of false positives added is 50% of the simulated set size. Results show that as the number of combined GOBP terms increases, the performance gap between ModGenePlexus and GenePlexus widens, with ModGenePlexus consistently outperforming GenePlexus, particularly for multi-term traits.

One challenge in designing and validating *ModGenePlexus* was the lack of a gold-standard disease module set (The DREAM Module Identification Challenge Consortium *et al.* 2019). To address this challenge, we constructed large gene sets (simulated “traits”) composed of biologically meaningful “modules” using sets of genes annotated to GOBP terms. Each trait consisted of either two, five, or ten GOBPs, treating each GOBP as an individual gene module. To mimic false positives and real-world noise, we introduced randomly sampled non-GOBP genes, where false positives were introduced such that the number of false positives is 0.25, 0.50, and 1.0 times the number of genes in the original set. *ModGenePlexus* consistently outperformed *GenePlexus* where performance was measured using log2(auPRC/prior) (see Section 2; Fig. 3, Fig. 1, available as supplementary data at *Bioinformatics* online).

As the number of combined GOBP terms increased and false positives grew, *ModGenePlexus’*s advantage over *GenePlexus* became more pronounced. The only exception was when testing a trait made of a singular GOBP—in which case *GenePlexus* performed better when no false positives were present, but both methods performed similarly as false positives were introduced. This outcome aligns with *GenePlexus’*s established ability in classifying genes for individual GOBPs. Overall, *ModGenePlexus* excels when analyzing datasets with multiple distinct biological processes or when false positives are prevalent.

### 3.4 *ModGenePlexus* outperforms the single-model approach in gene classification of transcriptomics-derived gene lists

We designed *ModGenePlexus* to handle real-world gene-based results from omics-scale experiments, the most common being large, noisy lists of differentially expressed genes (DEGs) from RNA-seq or microarray experiments. To test our method’s effectiveness in this setting, we evaluated *ModGenePlexus* on the DEG results of a manually-curated collection of public microarray experiments studying three types of “conditions:” diseases, gene perturbations, and drug treatments. These DEG gene lists pose an ideal challenge for *ModGenePlexus* because they often contain hundreds or thousands of gene associations and high levels of noise (Raser and O’Shea 2005, Eling *et al.* 2019). After filtering based on size and experiment count, we obtained 311 disease gene lists, 972 gene perturbation gene lists, and 234 drug treatment gene lists with ≥100 genes total and ≥10 understudied genes in the positive test genes (*see Materials and methods*). We first tested the full *ModGenePlexus* pipeline, which integrates module finding, denoising, semi-supervised gene expansion, and supervised gene classification (via *GenePlexus*).

**Figure 4:**
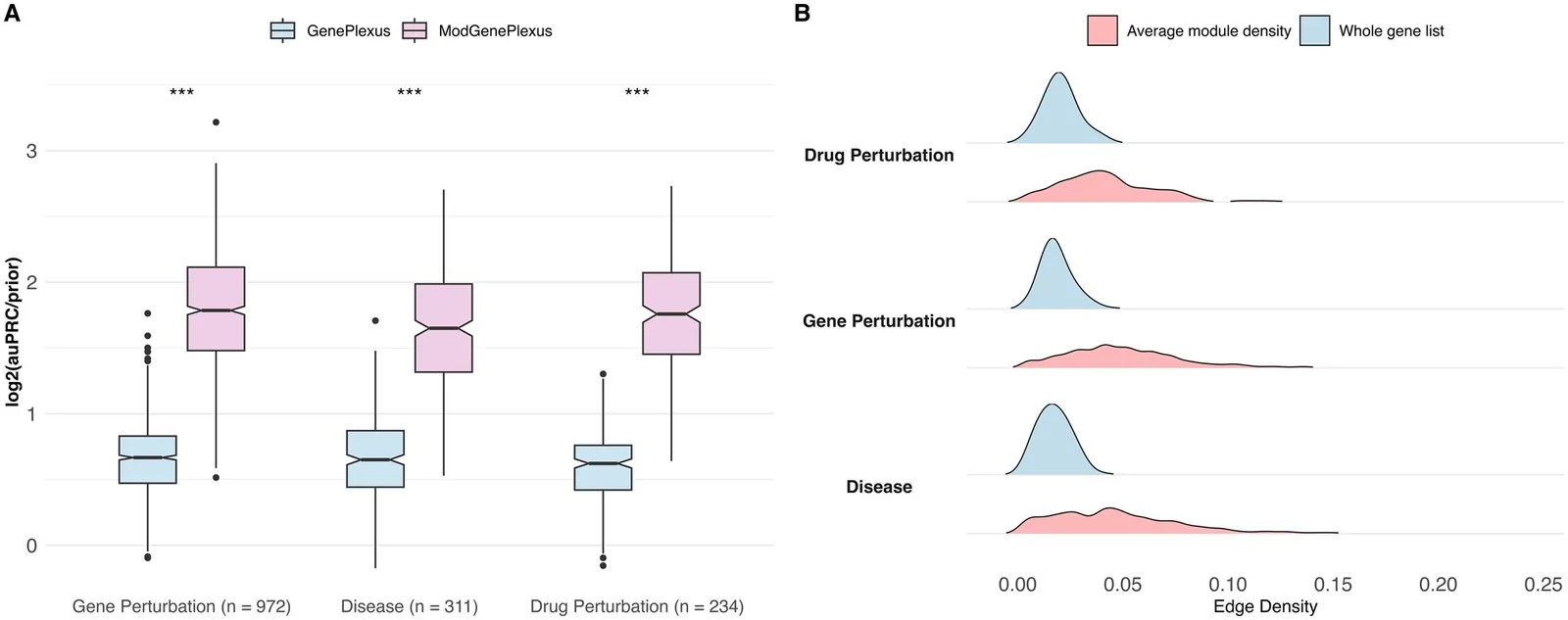
ModGenePlexus outperforms GenePlexus for transcriptomic gene lists. (A) ModGenePlexus significantly outperforms GenePlexus across three transcriptomic gene set collections from CREEDS: gene perturbations, disease, and drug perturbations. Stars refer to a Wilcoxon rank-sum test, where significance values are *P* = 6.49e − 295, *P* = 1.63e − 87, *P* = 5.08e − 75, respectively. (B) Distribution of network edge densities for GenePlexus input (whole gene set) and the average edge density for modules (Average module density). Module edge densities are typically higher than the edge density of the whole initial set.

**Figure 5:**
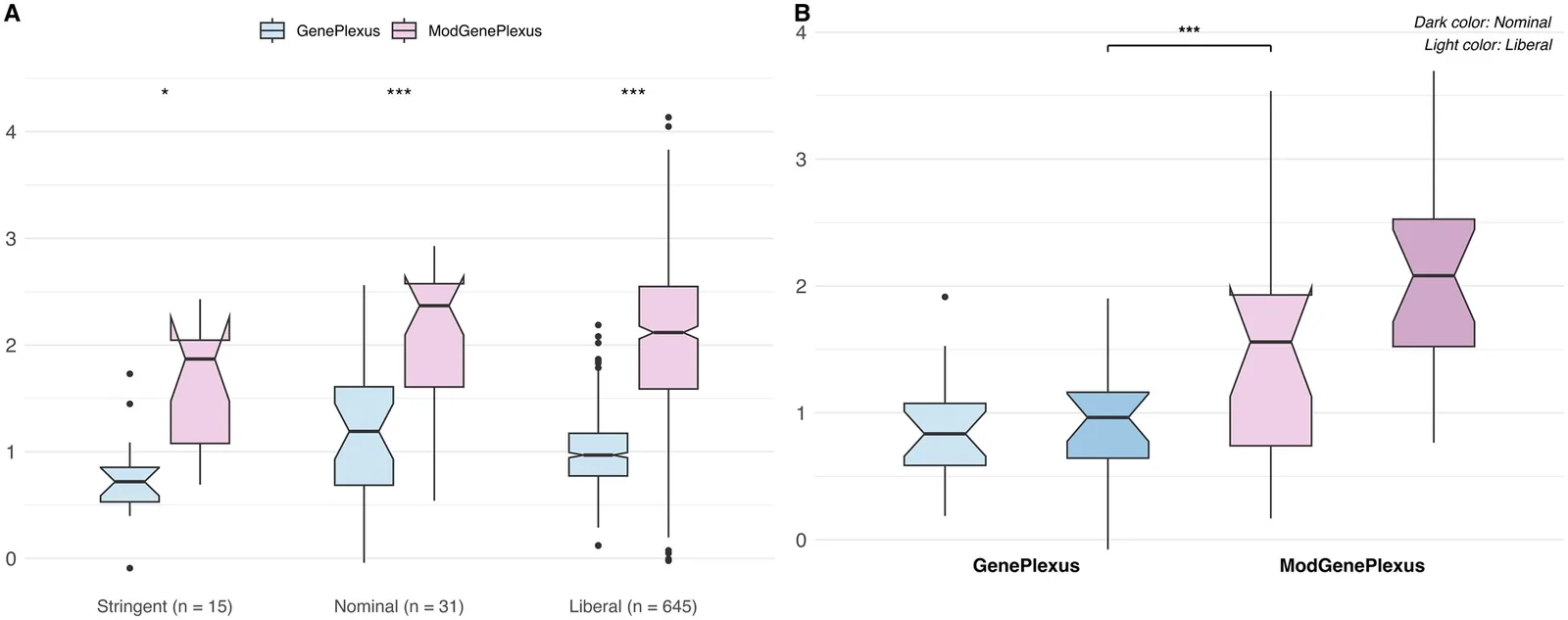
ModGenePlexus outperforms GenePlexus on GWAS gene level predictions. (A) Performance comparison using MAGMA-derived gene scores from GWAS summary statistics at three significance thresholds (Stringent: *P* < 1e − 8, Nominal: *P* < 1e − 5, and Liberal: *P* < 1e − 2). ModGenePlexus consistently outperforms GenePlexus across all thresholds, though both methods exhibit a decline in performance at the strictest threshold (Stringent). Stars refer to a Wilcoxon rank-sum test, where significance values are *P* = 1.32e − 4, *P* = 8.93e − 7, *P* = 1.01e − 131 respectively. (B) ModGenePlexus models trained on less stringent GWAS thresholds (Nominal, Liberal) demonstrate improved classification performance when evaluated on 1e − 5 test genes, outperforming the standard GenePlexus model where the original GenePlexus performance was low. Wilcoxon sum rank test *P* < .001.

Again, using *GenePlexus* as our baseline, across all types of conditions, *ModGenePlexus* significantly outperforms *GenePlexus* (Fig. 4A). This better performance holds even when we skip the semi-supervised gene expansion step, showing the importance of roughly breaking down large, complex gene lists into modules (Fig. 2, available as supplementary data at *Bioinformatics* online). We additionally calculated the edge density of the original gene set and the obtained modules. The average edge density of the modules is most often greater than the edge density of the original gene set used for *GenePlexus* (Fig. 4B), which confirms that the modules are more cohesive in the underlying network. We also tested the importance of the denoising step by pooling all genes contained in any module into a single gene list and then using that combined gene list to train a single *GenePlexus* model (Fig. 3, available as supplementary data at *Bioinformatics* online). We see that while pooling the denoised modules into a single gene list leads to some improvement to *GenePlexus*, its performance still falls way short of *ModGenePlexus*, reinforcing the advantage of analyzing individual modules separately rather than treating the gene list that contains multi-factorial biology as a single unit. Lastly we analyzed the performance of predicted *DOMINO* genes and compared to *ModGenePlexus* results. *DOMINO* failed to recover most positive genes and *ModGenePlexus* demonstrated superior classification accuracy (Figs 4 and 5, available as supplementary data at *Bioinformatics* online).

### 3.5 *ModGenePlexus* outperforms the single-model approach in gene classification of GWAS-derived gene lists

The second major application area for *ModGenePlexus* is to reprioritize and interpret trait- or disease-associated genes from a GWAS. Compared to analyzing the gene-based results of functional omics studies like transcriptomics, the application to GWAS presents unique challenges due to stringent multiple hypothesis corrections of summary statistics at the level of SNPs and genes (derived using algorithms like *MAGMA* (Leeuw *et al.* 2015)). These challenges intensify as GWAS sample sizes increase because more significant hits are constantly discovered (Wray *et al.* 2018). To assess the performance of *ModGenePlexus* on GWAS results, we applied it to gene-based summaries for a wide range of traits and diseases generated by *MAGMA*, compiled in GWAS Atlas (Tian *et al.* 2020). We used three significance thresholds (liberal, nominal, and stringent)—$P<1\times 10^{-2},\;P<1\times 10^{-5}$, and $P<1\times 10^{-8}$—to derive gene lists associated with each trait/disease and retained gene lists with both ≥100 genes and statistically significant modules returned from *DOMINO*, which resulted in 645, 31, and 15 traits/diseases for each threshold, respectively.

For all three thresholds, *ModGenePlexus* outperformed *GenePlexus* (Fig. 5A). Notably, *GenePlexus* performed considerably worse on GWAS data with the most stringent threshold, often yielding performance worse than random. In an alternative validation approach, we trained models using genes defined at the liberal and nominal thresholds ($P<1\times 10^{-2}$ and $P<1\times 10^{-5}$) but evaluated them on understudied genes defined at the nominal $P<1\times 10^{-5}$ threshold. These two thresholds were selected because they yielded enough sufficiently-sized gene lists to support robust model training and evaluation. We also focused on the traits where the original *GenePlexus* performance was poor (log2(auPRC/prior) <2). The $P<1\times 10^{-5}$  *ModGenePlexus* model performed best (Fig. 5B), with the $P<1\times 10^{-2}$ model also surpassing *GenePlexus* when evaluated at $P<1\times 10^{-5}$. These findings show that *ModGenePlexus* leverages module discovery to filter out poor-quality hits more effectively than using a strict *P*-value threshold to compile a gene list. By reducing the impact of false positives while retaining biologically relevant genes, *ModGenePlexus* enhances GWAS-based gene classification, particularly for understudied and less significant genes.

### 3.6 *ModGenePlexus* intrinsically learns local network neighborhoods and provides substantially interpretable enrichment results

**Figure 6:**
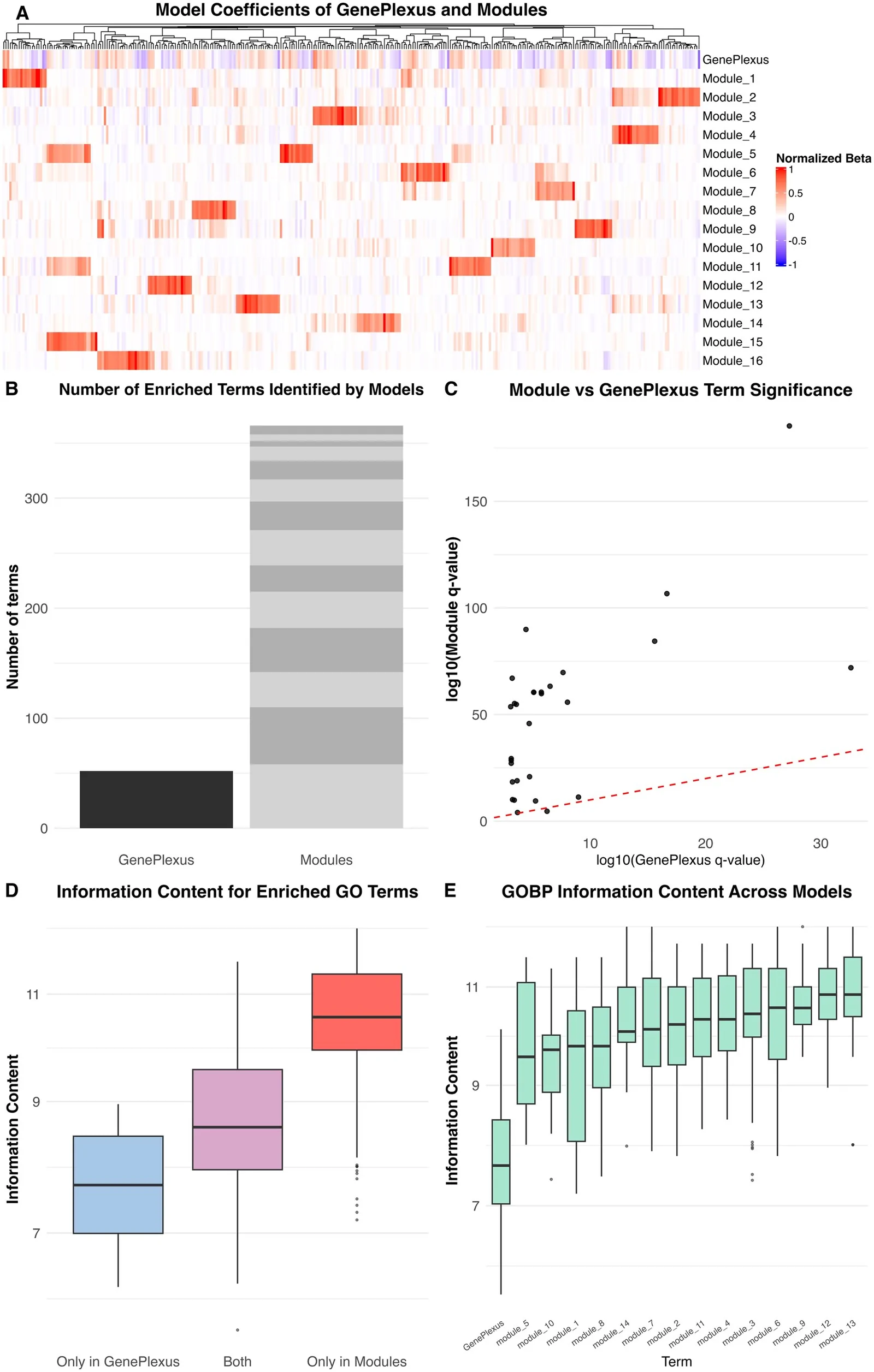
Using modules uncovers relevant biology that a full gene set misses. (A) Normalized logistic regression model coefficients (min-max scaled) for GenePlexus and ModGenePlexus. ModGenePlexus modules exhibit more concentrated weight values within trait-specific modules, whereas GenePlexus coefficients are more diffuse and less significant. (B) Number of enriched GOBP terms identified by GenePlexus and ModGenePlexus. The module-based approach results in substantially more enriched terms compared to GenePlexus. (C) Enrichment values of GOBP terms shared between GenePlexus and ModGenePlexus. Module-level enrichment values in ModGenePlexus are significantly higher than those observed in GenePlexus predictions based on the entire gene list. (D) Information content distribution of enriched GOBP terms categorized by enrichment in only GenePlexus, only ModGenePlexus, or both. Terms enriched in both methods, as well as those enriched only in GenePlexus, have smaller information content than those enriched exclusively in ModGenePlexus, reflecting its finer biological resolution. (E) Comparison of enriched GOBP information content between GenePlexus and ModGenePlexus modules. Module-based enriched terms have higher information content than those identified using the entire gene set.

To confirm *ModGenePlexus’*s ability to isolate specific signals within large gene lists, we examined the model’s learned coefficients which offer insight into the network-based signals driving its predictions. We visualized the beta coefficients from the supervised learning models created in *GenePlexus* and *ModGenePlexus* to determine if beta coefficient values were more extreme or more specific in *ModGenePlexus* models compared to *GenePlexus*. *ModGenePlexus* highlighted smaller, high-confidence gene subsets which suggests context-specific biological relevance. In contrast, *GenePlexus* distributed lower-weighted coefficients across a broader gene list (Fig. 6A).

The primary approach for interpreting long gene lists from high-throughput studies is to perform gene set enrichment analysis to identify overrepresented pathways/processes, which may be relevant to the condition being studied. Therefore, we decided to assess the impact of gene classification and reprioritization by *ModGenePlexus* on enrichment-based biological interpretability. To do so, we compared the results of GO Biological Process (GOBP) enrichment on the top-ranked genes from *ModGenePlexus* and *GenePlexus*, using Type 2 diabetes as a case study. This disease has a highly heterogeneous signature, with ∼2000 genes across 14 CREEDS-derived differential expression studies. *ModGenePlexus* improved gene classification performance, prompting further analysis of downstream enrichment. Multiple correction was performed (Benjamini-Hochberg; P<.001) to determine significance. *ModGenePlexus* leads to recovering significantly more enriched terms than *GenePlexus*, including many captured only by *ModGenePlexus* (Fig. 6B). Furthermore, terms shared between both methods show stronger enrichment under *ModGenePlexus* (Fig. 6C), suggesting higher specificity and precision. For example, GOBP cellular response to insulin was discovered by *ModGenePlexus* ($\text{BH}=4.10\times 10^{-6}$) but not *GenePlexus* ($\text{BH}=4.63\times 10^{-1}$). To test whether *ModGenePlexus* yields more focused biological insights, we compared the size of enriched gene lists. Module-level enrichment from *ModGenePlexus* produced more compact, functionally distinct terms (Fig. 6D and E), while *GenePlexus*-enriched terms had less information content. Terms unique to *GenePlexus* also had less information content than those shared between methods (Fig. 6C). Shared terms also tended to have higher information content than those enriched exclusively in *ModGenePlexus* results. In total, *ModGenePlexus* yielded 366 unique enriched terms compared to 52 from *GenePlexus* (Fig. 6, available as supplementary data at *Bioinformatics* online), and some terms such as oxidative phosphorylation were enriched across multiple modules (Table 1, available as supplementary data at *Bioinformatics* online) suggesting that modules with distinct genes work together in the same or similar pathways. Together, these observations indicate that *ModGenePlexus* enhances interpretability by emphasizing high-resolution, modular signals rather than high-level associations seen in typical enrichment analysis of gene-based outputs from high-throughput experiments. To explore these differences further, we mapped the top-ranked predictions from both *GenePlexus* and *ModGenePlexus* to discover relevant and diverse processes discovered by *ModGenePlexus* but not *GenePlexus*, displayed in enrichment tables (Tables 1–6, available as supplementary data at *Bioinformatics* online).

## 4 Discussion

Improved prioritization of trait/disease-associated genes from large-scale experimental datasets using genome-scale gene networks presents significant challenges due to gene set size, noise, and biological complexity. Standard approaches, including label propagation (semi-supervised learning) and *GenePlexus* (supervised learning), that treat the entire gene list as a single unit struggle to classify genes in such cases (Liu *et al.* 2020b), necessitating new methods. Here, we present *ModGenePlexus*, a novel approach that addresses key limitations in network-based supervised learning with large, noisy, heterogeneous gene lists by leveraging modular structure present in gene networks, filtering out poorly connected genes (likely false positives), and producing not one but multiple reprioritized gene lists, each corresponding to network-defined biologically coherent biological processes. While several existing gene reprioritization methods (Fine *et al.* 2019) generate a single genome-wide ranking of genes for a trait or disease, this single output can be difficult to interpret in practice. Given the high polygenicity of complex traits/diseases, important disease-associated genes may appear far below the top of such rankings—well beyond the intuitive cutoffs researchers typically consider for follow-up (e.g. top 10, 50, or even 100 genes). Furthermore, these single-list outputs still require manual interpretation of individual genes or post-hoc grouping based on pathway enrichment. Because in *ModGenePlexus* each gene list is explicitly linked to a network module and the corresponding enriched pathways, interpreting these results for follow-up studies becomes more straightforward. Thus, *ModGenePlexus’*s modular outputs are able to substantially elevate the interpretability and practical utility of the gene prioritization results.

We demonstrated that *GenePlexus* performs poorly on large experimental gene sets, particularly those from transcriptomic studies, where high noise and poor network localization weaken classification accuracy of single-model methods. Because *GenePlexus* applies supervised learning on the full original gene set, it cannot function as a standalone solution for these datasets. This limitation underscores a fundamental limitation of network-based classifiers: their reliance on globally coherent patterns is incompatible with the heterogeneous biological reality of polygenic traits. We showed that *ModGenePlexus’*s advantage grows as gene sets increase in size and contain more false positives. As the number of distinct perturbed pathways and processes increases, *ModGenePlexus* effectively separates distinct biological modules and improves gene classification in more complex traits. *ModGenePlexus* significantly improves gene classification for results from transcriptomic datasets. DEG lists from such datasets are not only large but also highly noisy, lacking strong network localization. By integrating filtering, semi-supervised propagation, and supervised learning, *ModGenePlexus’*s ability to filter noise and propagate meaningful gene associations makes the input gene set more topologically and biologically coherent and enables more biologically meaningful predictions for real omics data. Furthermore, we demonstrated that *ModGenePlexus* predictions are not driven by hub gene connectivity; high-degree genes are ranked less favorably on average, confirming that *ModGenePlexus* recovers biologically meaningful genes rather than simply prioritizing well-connected network hubs (Figs 7 and 8, available as supplementary data at *Bioinformatics* online).

We use *DOMINO* as the method to cluster the large input gene list into modules. In this step, *DOMINO* also “denoises” the gene list using an approach akin to semi-supervised learning, dropping genes that are not strongly connected and including new genes in the network that are critical for the connectivity of the remaining genes. We demonstrate that using this semi-supervised learning step improves *ModGenePlexus* results and that this improvement occurs by just using the module-based breakdown of the original genes even without the newly-included genes (Fig. 2, available as supplementary data at *Bioinformatics* online). The broader motivation for decomposing omics-derived gene lists into distinct network modules is shared by other network-based approaches. Therefore, though we used *DOMINO* in this study, in principle, any network clustering method that partitions a large input gene list into optimal modules (The DREAM Module Identification Challenge Consortium *et al.* 2019, Yang *et al.* 2024) can be used in *ModGenePlexus*.

Similar to its performance on gene lists derived from transcriptomics studies, *ModGenePlexus* enhances gene reprioritization for GWAS datasets. We show that training the model with genes selected based on nominal *P*-value thresholds improves the performance compared to training on genes from liberal thresholds. On the other hand, training on genes from the strictest threshold ($P<1\times 10^{-8}$) resulted in comparatively lower performance. This performance decrease likely results from the small number of retained genes that represent disparate pathways and processes involved in the trait/disease (i.e. one or a couple of genes from most of the relevant pathways rather than multiple genes from any one pathway), meaning they are unlikely to be close together in the underlying network. When the threshold is relaxed to a nominal value, other genes in relevant pathways start entering the initial list and coalesce around the top genes in the underlying network, forming more cohesive modules that *ModGenePlexus* can leverage. Previous studies demonstrate that genes with more nominal *P*-values can still have biological relevance (Chen *et al.* 2021), meaning strict thresholds may remove false positives but also introduce false negatives. Solely relying on strict genome-wide significance thresholds may systematically underrepresent functionally important genes. *ModGenePlexus’*s network-based refinement offers a principled solution to this longstanding challenge in GWAS interpretation.

Beyond superior performance in reprioritizing genes, using Type 2 Diabetes as a case study, we show how *ModGenePlexus* can lead to greater biological insights compared to *GenePlexus*. *ModGenePlexus* uncovers more granular biological pathways (Fig. 6) by leveraging modular network structure and the ability to use module-specific model results, rather than the aggregation into a single list. For example, from the list of genes differentially expressed in Type 2 Diabetes, *ModGenePlexus* uniquely identified enrichment in copper ion transport (Gong *et al.* 2008, 2009, Tanaka *et al.* 2009, Gembillo *et al.* 2022) in module 5 (Table 2, available as supplementary data at *Bioinformatics* online) and iron homeostasis pathways (Liu *et al.* 2020a, Ma *et al.* 2021, Feng *et al.* 2023, Miao *et al.* 2023) in modules 1 and 3, which the single-model approach failed to detect (Tables 3–5, available as supplementary data at *Bioinformatics* online). Distinct insulin-related (Brännmark *et al.* 2013, Boucher *et al.* 2014, Dall’Agnese *et al.* 2022) pathways (enriched in modules 7 and 12) were only detected by *ModGenePlexus* (Table 5, available as supplementary data at *Bioinformatics* online). These findings illustrate how *ModGenePlexus* reveals biological processes that would be overlooked by considering all disease genes as a single unit.

A few limitations of this study and gene classification methods warrant consideration. First, gene interactions are highly context-dependent, varying across tissues, cell types, and disease states, and thus the performance of any network-based analysis will not only depend on the underlying network properties such as density and coverage, but also the context-specific information used to construct it. A general-purpose network such as STRING may obscure condition-specific interactions or bias module detection. However, we chose a general network deliberately because systematic benchmarking across diverse biological settings requires a consistent foundation, and no gold standard context-specific network exists that spans the range of traits and diseases we evaluated. Matching each trait/disease with the “right” context-specific network is another challenge. Evaluating *ModGenePlexus* with context-specific networks [like GIANT (Wong *et al.* 2018) and CONE (Liu *et al.* 2023) generated by our group] constitutes important future work.

## 5 Conclusion

*ModGenePlexus* provides a scalable and biologically meaningful approach to prioritize trait/disease-associated genes from large, noisy, heterogeneous gene lists derived from high-throughput experiments. By leveraging modular network structure and integrating filtering, propagation, and supervised learning, *ModGenePlexus’*s divide-and-conquer approach significantly outperforms single-model methods like *GenePlexus* in network-based prioritization of genes from transcriptomic and GWAS datasets. Ultimately, its ability to refine gene associations and uncover biologically meaningful pathways makes *ModGenePlexus* a powerful tool for post-omics hypothesis generation across diverse disease studies.

## Supplementary Material

btag571_Supplementary_Data

## Data Availability

The data used in this study, including the gene list collections and networks, are freely available on the GitHub repository *ModGenePlexus*, https://github.com/krishnanlab/ModGenePlexus, which contains code to reproduce the results presented in this study. The full source code and results supporting this study are publicly available on Zenodo at https://zenodo.org/records/19857910.
