# Supplementary material for: A module-based approach for post-omics, post-GWAS network-based gene classification
Source: Bioinformatics. 2026 Jul 31;42(8):btag571. doi: 10.1093/bioinformatics/btag571 (PMC13524082; doi:10.1093/bioinformatics/btag571)
Supplement: Supplementary file 1 [file btag571_supplementary_data.zip › ModGenePlexus_Supplemental.docx]

**Supplement: A module-based approach for post-omics, post-GWAS network-based gene classification**

**Figure S1:** ***ModGenePlexus* outperforms *GenePlexus* on simulated traits generated by combining multiple GOBP terms.** *Performance on simulated traits generated by combining multiple GOBP terms. Models were tested on individual GOBP terms and simulated traits composed of 2, 5, and 10 combined terms. False positives were introduced at rates of 0, 0.25, 0.50, 0.75, and 1.0, corresponding to proportions of the original set size. Results show that as the number of combined GOBP terms increases, the performance gap between ModGenePlexus and GenePlexus widens, with ModGenePlexus consistently outperforming GenePlexus, particularly for multi-term traits.*

**Evaluating additional model steps between *GenePlexus* and *ModGenePlexus***
*ModGenePlexus* introduces several key differences compared to *GenePlexus*. with the most notable being the incorporation of a filtering step during module discovery. This step removes genes that do not belong to sufficiently large modules, ensuring that only robust modules are used for training logistic regression models. Additionally *GenePlexus* is used on top of DOMINO-derived modules. This module discovery process is itself a form of gene classification as genes are propagated that were not part of the initial geneset. Lastly, *ModGenePlexus* creates multiple models – one for each module – rather than a single model for all module genes. We evaluated our method in multiple steps of this pipeline for the transcriptomic datasets. First we run *ModGenePlexus* on modules without their propagated genes, except the propagated positive test genes are included. By including these we tested whether *GenePlexus* is able to distinguish between positive and negative labels better by using the neutral propagated genes, or if *GenePlexus* is only performing better because the semi-supervised learning in DOMINO successfully recovered some positive test genes. We demonstrate that using all propagated genes from DOMINO gives superior results for *ModGenePlexus* (**Figure S2A)**. We further show that the increase in performance is not solely driven by the incidental propagation of initial positive test genes (**Figure S2B**). We next assessed a baseline where a single model is created for all module genes – those that are part of the initial disease, in big enough modules, and the propagated genes – rather than creating an individual model for each module. Our results indicate that *ModGenePlexus* significantly outperforms this approach when individual models are created for each module (**Figure S3**).

**Figure S2: Full *ModGenePlexus* outperforms *ModGenePlexus* without propagation.** *(A) These plots compare the performance of GenePlexus (blue), ModGenePlexus (pink), and ModGenePlexus without propagation (green). ModGenePlexus performance when not including genes propagated from DOMINO clustering performs worse than the full ModGenePlexus method. (B) Removing only the propagated genes that are known held-out test positives from the supervised label set does not significantly affect performance, in contrast to the full removal of propagated labels shown in panel A.*

**Figure S3: Full *ModGenePlexus* outperforms *GenePlexus* on an individual list of all module genes.** *GenePlexus performance when only using genes that were assigned to a large enough module (>= 10 genes) in DOMINO is inferior to ModGenePlexus*

**Evaluating gene classification with DOMINO vs *ModGenePlexus***

The DOMINO clustering algorithm performs a form of gene classification by propagating genes that are strongly connected to discovered modules. This raises an important question: do *GenePlexus* and *ModGenePlexus* provide added utility beyond what DOMINO alone contributes? To address this, we compared the performance of DOMINO-based gene classification to *ModGenePlexus* by calculating F1 scores using two complementary approaches. First, we computed the F1 score at the maximum point on the auPRC curve **(Figure S4A)**, providing an optimistic view of model performance. Second, we computed F1 scores using the top N predictions from *ModGenePlexus*, where N equals the number of genes propagated by DOMINO after clustering **(Figure S4B)**, providing a stricter and more conservative evaluation. Two evaluations are also useful because it is user-decision what genes to use from *(Mod)GenePlexus* results. To calculate DOMINO’s F1 scores, we labeled the propagated test genes – both positive and negative – and calculated precision and recall accordingly. Across both evaluation strategies, *ModGenePlexus* consistently outperformed DOMINO for the majority of transcriptomics disease gene sets. The performance gap was especially pronounced when evaluating F1 at the auPRC maximum, but even under the more conservative top-N condition, *ModGenePlexus* still demonstrated superior classification accuracy.

**Figure S4: Comparing F1 scores of DOMINO and *ModGenePlexus* for transcriptomics disease gene sets.** *(A) F1 scores are calculated at the optimal point on the auPRC curve, representing the best-case performance of ModGenePlexus. (B) A more conservative evaluation is shown, where the number of predicted genes from ModGenePlexus is matched to the number of genes propagated by DOMINO. In both settings, ModGenePlexus consistently outperforms DOMINO-based gene classification.*

**Limitations of DOMINO gene classification**

A key advantage of *ModGenePlexus* is that it provides predictions for all genes in the network, allowing users to assess genome-wide relationships to their input gene list. In contrast, DOMINO is a binary classification algorithm, meaning it only outputs propagated genes while discarding those removed during clustering. This limits its ability to offer a comprehensive gene classification framework. **Figure** **S5A** illustrates that for the vast majority of transcriptomics disease datasets, fewer than 10% of positive test genes are recovered during DOMINO’s propagation step. Additionally, **Figure** **S5B** shows that when examining the composition of propagated genes, negative test genes outnumber positive test genes in most cases. This suggests that DOMINO not only fails to recover many disease-associated genes but also propagates a substantial number of negative test genes, reducing classification precision. Furthermore, its lack of genome-wide predictions prevents users from fully contextualizing their input gene sets. These limitations highlight a major advantage of *(Mod)GenePlexus*, which retains genome-wide interpretability while improving classification accuracy.

**Figure S5: Domino gene classification alone fails to uncover most known positive genes.** *(A) Proportion of positive test genes recovered by DOMINO relative to the total number of positive test genes in transcriptomics disease datasets. (B)* *Proportion of positive test genes among all test genes propagated by DOMINO, showing that negative test genes often outnumber positives.*

**Figure S6:** **The number of enriched GOBP terms in *GenePlexus* and modules.** *Enrichment on modules uncovers many more terms than running enrichment on the full initial gene list.*

**Modularity of GO terms used in simulations**

GO Biological Process terms used in the combination simulations were pre-filtered for non-redundancy using Jaccard and overlap index thresholds to ensure representative term selection (for a detailed description see Liu et al.). To verify that randomly sampled GO term combinations are composed of effectively distinct modules, where each sampled GO term represents a discrete biological process, we characterized the pairwise gene overlap across the filtered GO term set.

Pairwise Jaccard similarity was computed across all GO term pairs in the dataset. The median pairwise Jaccard was 0, with 73.6% of pairs sharing no genes and 99.4% of pairs having a Jaccard below 0.1. For the combination experiments, the expected mean pairwise Jaccard within randomly drawn sets of 2, 5, or 10 terms was approximately 0.005 in all cases. These results confirm that the simulated multi-module traits are composed of largely non-overlapping gene sets.

**Assessment of hub node bias in GenePlexus predictions**

A potential concern with network-based gene prioritization methods is that highly connected hub genes may receive inflated prediction scores simply due to their network topology rather than true disease relevance. We addressed this concern in two ways. First, we examined whether the study-bias train/test split design structurally mitigates hub bias. Because well-studied genes tend to have higher network degree, the study-bias split intentionally places high-degree genes into the training set, leaving understudied and predominantly low-degree genes as the held-out evaluation set. To verify this empirically, we compared the degree distributions of training versus test genes across all CREEDs diseases in the STRING network. Training genes showed substantially higher network degree than test genes (Wilcoxon test, p < 2.2e-16; **Figure S7**), confirming that hub genes are captured as known positives rather than appearing as potential confounders in evaluation.

Second, we directly assessed whether GenePlexus predictions are inflated for high-degree genes. We computed the mean rank of all non-training genes across CREEDS diseases and binned genes by network degree into deciles. Contrary to a hub bias concern, genes in the highest degree decile (degree 148–1393) received systematically lower mean ranks than low-degree genes (**Figure S8**). This is consistent with the expectation that ubiquitously connected hub genes receive diluted disease-specific signal during training rather than artificially elevated scores.These analyses demonstrate that recovery of true positives is not driven by hub gene connectivity, and that the evaluation framework is structurally protected from this form of bias by design.

**Figure S7:** **The distributions of training and test gene node degree**. Training genes have notably and significantly higher node degrees in the STRING network than test genes do.

**Figure S8:** **GenePlexus prediction rank is not inflated for high-degree hub genes.** Violin plots show the distribution of mean prediction rank for all non-training (U) genes, averaged across CREEDs diseases, binned into deciles by STRING network degree. The y-axis is such that rank 1 (highest priority) appears at the top. Genes in higher degree deciles receive systematically worse mean ranks, with the distribution shifting downward from D1 (degree 1–2) to D10 (degree 148–1393). Internal boxplots show the median and interquartile range per bin.

**Table S1:** GOBP terms enriched by both module 1 and module 3 for Type 2 Diabetes.

**Table S2**: The top 20 terms enriched for module 5 for Type 2 Diabetes. This includes the relevant copper ion transport pathway – enriched for module 5 but not for *GenePlexus*.

**Table S3:** The top 20 terms enriched by module 1 for Type 2 Diabetes. This includes the relevant iron-sulfer module assembly pathway – enriched for module 1 but not for *GenePlexus*.

**Table S4**: Iron-related pathways found enriched in modules 1 and 3 for Type 2 Diabetes.

**Table S5:** Insulin-related terms enriched in modules 7 and 12. These were enriched for multiple modules but not for *GenePlexus*.

**Table S6:** The top 20 GOBPs enriched by *GenePlexus* for Type 2 Diabetes.

**References**

1.[Liu, R., Mancuso, C. A., Yannakopoulos, A., Johnson, K. A. & Krishnan, A. Supervised-learning is an accurate method for network-based gene classification. *Bioinformatics* https://doi.org/10.1093/bioinformatics/btaa150 (2020) doi:10.1093/bioinformatics/btaa150.](https://www.zotero.org/google-docs/?zpGT8P)
